# plaTform fOr Urinary tract infection diagnostiC evAluatioN (TOUCAN): a protocol for a prospective diagnostic accuracy study of point-of-care testing in patients suspected of acute uncomplicated urinary tract infection in primary care clinics in England

**DOI:** 10.1136/bmjopen-2024-090012

**Published:** 2025-01-30

**Authors:** Philip J Turner, Thomas R Fanshawe, Jane Freeman, Margaret Glogowska, Alastair D Hay, Nicola Kenealy, Owain Llion, Rebecca Lowe, Mark Lown, Michael Moore, Valerie Tate, Mark H Wilcox, Mandy Wootton, Christopher C Butler, Gail N Hayward

**Affiliations:** 1 Nuffield Department of Primary Care Health Sciences, University of Oxford, Oxford, UK; 2 University of Leeds, Leeds, UK; 3 Centre for Academic Primary Care, University of Bristol, Bristol, UK; 4 Primary Care and Population Sciences, University of Southampton, Southampton, UK; 5 Primary Care Medical Group, University of Southampton Medical School, Southampton, UK; 6 Public co-investigator – unaffiliated, Oxford, UK; 7 Leeds Teaching Hospitals & Leeds Institute of Medical Research, University of Leeds, Leeds, UK; 8 Specialist Antimicrobial Chemotherapy Unit, Microbiology Cardiff, Public Health Wales, Cardiff University, Cardiff, UK

**Keywords:** Primary Care, Urinary tract infections, Diagnostic microbiology, Antibiotics

## Abstract

**Abstract:**

**Introduction:**

Acute uncomplicated urinary tract infection (UTI) is a common condition with potentially serious sequelae that is mostly diagnosed and managed in primary care settings. Around half of all women have a UTI in their lifetime, and a quarter experience an infection caused by organisms resistant to more than one antibiotic. Reducing inappropriate prescribing of antibiotics is a core tenet of antimicrobial stewardship. However, current diagnostics for UTI are unfit for purpose in acute (highest prescribing) settings, being too slow to inform the required immediate decision-making and often confounded by sample contamination.

Rapid point-of-care diagnostic tests (POCTs) that facilitate timely decision-making are potential solutions to this problem. Several such tests have reached advanced stages of technology readiness, but their diagnostic performance has not been evaluated in primary care with clinical users. To progress novel tests towards implementation, a diagnostic field study is required, to allow for parallel and sequential evaluation of multiple tests in a primary care population.

**Methods and analysis:**

We will recruit participants assigned female at birth from primary care clinics in England who contact their clinic with symptoms of acute uncomplicated UTI. Eligible participants will complete a short questionnaire to capture symptoms and symptom severity and will provide a urine sample. Samples will be split and initially tested using novel index tests (POCTs) and conventional urinalysis ‘dipstick’ at the primary care clinic. The second part of the sample will be processed at a National Health Service-based reference laboratory using a modified reference standard including microscopy, microbiological culture, pathogen speciation and antimicrobial susceptibility testing. The UTI reference standard culture, although based on the national methods, is modified to provide accurate bacterial counts, better to define a microbiological diagnosis of UTI. Susceptibility testing will be performed using ‘gold-standard’ methods, not usually performed in diagnostic laboratories. The primary outcome will be the diagnostic performance (sensitivity, specificity, positive and negative predictive values) of POCTs for detection of UTI and antimicrobial susceptibility for POCTs that include antimicrobial susceptibility testing. Secondary outcomes will include the symptom profile of patients presenting with uncomplicated UTI, a theoretical determination of how use of POCT results might change prescribing, an understanding of POCT failure rate and qualitative capture of the experiences of those using the POCT to deliver the study in primary care clinics.

**Ethics and dissemination:**

Ethical approval was received from the London Central Research Ethics Committee (23/LO/0371) and the UK Health Research Authority. We will publish the findings of The plaTform fOr Urinary tract infection diagnostiC evAluatioN evaluations in peer-reviewed medical journals and more broadly following a dissemination plan formulated by a communications specialist in consultation with patients and the public.

**Trial registration number:**

ISRCTN80937472.

**STRENGTHS AND LIMITATIONS OF THIS STUDY**

- Prospective study design.
- Common sample used for (potentially) multiple index point-of-care tests (POCTs) and reference standard testing.
- Embedded qualitative study to capture clinical user perspectives on test use and implementation.
- Limited power to determine the diagnostic performance of POCTs that include antimicrobial susceptibility testing against all potential antibiotics included on POCT testing panels, due to low prevalence of organisms resistant to antibiotics such as nitrofurantoin.
- The modified urinary tract infection reference standard culture (as performed by the National Health Service-based reference laboratory), while being modified for improved accuracy still has limitations which may make interpretation of discordant results challenging—conducting multiple POCT simultaneously may help mitigate this.

## Introduction

 Half of all women experience a urinary tract infection (UTI) in their lifetime.^1^ The reference standard test for UTI is laboratory microscopy, microbiological culture and antimicrobial susceptibility testing (AST). This process typically takes 2–3 days and may not produce clinically useful information; up to 30% of samples can be contaminated by host flora^2^ and loss of bacterial viability or overgrowth with time can yield misleading or no results. Use of sample collection tubes containing boric acid mitigates against bacterial overgrowth, but use of these tubes has not been universally adopted by laboratories. In consequence, the majority of suspected infections encountered in primary care are identified and treated empirically using clinical criteria, which have limited sensitivity and specificity.^3^

Antimicrobial resistance is one of the greatest threats to human health and is driven further by inappropriate prescriptions of antibiotics.^4^ Our study of patients with microbiologically confirmed UTI in four EU member states detected urinary pathogens with resistance to a single antibiotic in 27% (57/209) of patients and resistance to >1 antibiotic in 25% of patients (53/209).^5^ Therefore, there is pressure to reduce inappropriate (unnecessary or incorrect) antibiotic prescribing for UTI, while acknowledging that undertreatment has the potential to lead to serious sequelae.^6^ The current standard of care involves using a combination of symptoms, signs and simple dipstick results to predict which women are most likely to have infection.^7^ Better tests are required to help clinicians avoid prescribing antibiotics in women who do not need them and prescribe the correct antibiotics to those who do.

Rapid point-of-care diagnostic tests (POCTs) are potential solutions to this problem. A survey of 1109 UK general practitioners (GPs) in 2012 highlighted UTI as the condition for which a POCT would be most helpful to support diagnosis.^8^ Current POCTs for UTI are suboptimal, with urine dipsticks neither sensitive nor specific enough^9^ and are unable to provide information on the antibiotic susceptibility of pathogens. Diagnostic tests based on point-of-care bacterial culture on solid media are available, but these incorporate a 12–24 hour incubation step and are therefore too slow to influence immediate decision-making, and a trial incorporating this approach showed minimal impact on appropriate antibiotic prescribing.^10^ Thus, improved and more rapid POCTs are needed.

New technologies are aiming to deliver a rapid diagnosis and, in some cases, a uropathogen AST at the point-of-care, facilitating timely and targeted treatment. Although a number of developers have carried out laboratory evaluation work using bacterial cultures, urine samples ‘spiked’ with known uropathogens, and genuine patient samples, there have been no robust independent evaluations of these exciting technologies in real-world settings. It is vital that the performance of these diagnostic tests is evaluated before these devices are considered for inclusion in interventional studies or clinical practice.

In addition to POCT technologies, which are at or close to clinical readiness and so fully powered evaluations are appropriate, there are a number of innovators with potentially transformative early-stage technologies that require crucial proof of principle evidence, using fresh urine samples, to proceed further with development. Under these circumstances, we intend to nest small pilot studies alongside our full-scale evaluations. New POCTs will be introduced into this platform study as and when available.

## Methods and analysis

### Study design and setting

The plaTform fOr Urinary tract infection diagnostiC evAluatioN (TOUCAN) is a prospective diagnostic accuracy study allowing for parallel evaluation of index POCTs (referred to henceforth as POCTs) in a consecutively enrolled primary care population of female patients ≥18 years of age presenting with symptoms of uncomplicated UTI. TOUCAN began recruitment in September 2023. New POCTs will be introduced into the study as and when they become available, following due consideration of recruitment timelines to reach sample size. Participants recruited from primary care clinics in England will be asked to provide a single urine sample which will undergo analysis by POCTs at the point-of-care alongside conventional urinalysis dipstick test, then reference standard laboratory urine processing including microscopy, modified culture and AST (performed using ‘gold standard’ methods) at the National Health Service (NHS)-based reference laboratory. Participants will provide the urine sample at baseline (the day they consult healthcare) and answer a short questionnaire including their age, ethnicity, symptoms and confirmation of eligibility criteria. There will be no further patient follow-up, as this is a diagnostic accuracy study not concerned with effect on participant outcomes. Given the observational nature of the study, and uncertainty about test accuracy at this stage, POCT results will not be shared with participants or used to support clinical decision-making.

### Primary objective

To determine the diagnostic performance in a primary care field study of novel POCTs for diagnosing UTI against standard microbiology laboratory processing, including ‘gold-standard’ AST results. The reference standard for the study will conform to the definitions of UTI detailed in the UK Health Security Agency guide on the investigation of urine^11^ to determine microbiological diagnosis of UTI, while antimicrobial susceptibilities will be assessed in accordance with the International Standard 20776-1.^12^ Outcome measures will include sensitivity, specificity, predictive values and likelihood ratios for the detection of UTI and for AST results where this is an element of the POCT.

### Secondary objectives

- To record the different symptoms of people who seek help from their GP with suspected uncomplicated UTI.
- Understand whether and how using the results of a new POCT would result in changes in antibiotic prescribing.
- Determine how often POCTs fail, and how frequently they give uninterpretable results.
- To explore the experiences and perceptions of primary care staff involved in the use of new POCT for UTI, and how these might affect the feasibility of future implementation.

### Study participants and their selection

People assigned female at birth, aged 18 years and over, presenting to primary care with suspected uncomplicated UTI will be eligible for participation in the study. The inclusion criteria detailed in box 1 are broad in order to encompass the widest range of inclusion criteria scenarios detailed across multiple manufacturers’ intended use statements.

Box 1 **Study inclusion and exclusion criteria**

Inclusion criteria

1. Female* (including pregnant women).
2. Aged ≥18 years.
3. Presenting to UK primary care with current symptoms that have been present for fewer than 7 days, that the patient or their primary care health professional considers are consistent with an uncomplicated UTI.
4. Clinician confirms that urine sample for analysis is useful for patient’s care.**
5. Participant is willing to give consent for participation in the study.

Exclusion criteria

1. Previously recruited to this study.
2. Unable to provide a sample that was taken within the timeframe specified by POCT developers.
3. Unable to understand and complete trial materials in English.

*Participants will be included only if they were assigned female at birth.

**For example, urine sample tested before antibiotics are started to guide decision to prescribe and nature of prescription, or sample tested where UTI symptoms have persisted despite antibiotics to guide decision to prescribe and nature of prescription.

POCT, point-of-care test; UTI, urinary tract infection.

### Recruitment

Study sites will be selected with the help of the National Institute for Health and Care Research Clinical Research Network and will consist of primary care practices that are willing and able to adhere to the requirements of the study protocol. Participants will be selected through participating practices and will be identified through two key routes:

- When they book and attend (in person or on the telephone) a clinical appointment for a suspected, uncomplicated UTI.
- When they submit a urine sample for testing for a suspected, uncomplicated UTI at their primary care practice.

Potential participants who call the practice and state they have a possible UTI will be asked to come to the practice to provide a sample, once at the practice they will be asked if they are happy to take part in the study and if they indicate they are, the recruitment process described below will be followed.

### Screening and eligibility assessment

This will be a prospective opportunistic-recruitment study. Sequential potential participants will be screened as and when they present to their primary care practice with a suspected UTI. Once the participant has read the participant information sheet (PIS), signed the participant summary sheet (PSS) to indicate their consent and completed the baseline questionnaire, their responses to the eligibility questions will be assessed and confirmed by the member of the practice team who will run the tests. Relevant sections of the participant’s medical records will be reviewed by an authorised member of practice staff and any medical conditions relevant to the performance of the POCTs will be recorded in the study database. Potential participants should either receive the PIS in person at the practice, or they can be sent a link via text message by the GP to the trial website where they should download the PIS before they attend the practice. Once they attend the practice to produce their urine sample they will be presented with the PSS/consent form to sign.

### Informed consent

Online or written versions of the PIS will be presented to the participants detailing no less than: the exact nature of the study; what it will involve for the participant; the implications and constraints of the protocol; the known side effects and any risks involved in taking part. As all patient data will be collected at the baseline visit, it will not be possible to withdraw data and samples from the study after the baseline assessments are completed. This will be clearly explained in the PIS. Potential participants will be given time to consider the information, and the opportunity to question the Investigator, their GP or other independent parties to decide whether they will participate in the study. If they agree, consent proportionate to the study type and risk will be given by the participant signing and dating the appropriate part of the PSS/consent form. Study sites will use a web-based database system to record eligibility and confirm participant entry into the study, prior to the samples being processed for research.

### Data collection

The study will use a bespoke study database with eligibility screening, symptom questionnaire, baseline assessment, POCT and laboratory electronic case report forms (eCRFs) developed on the REDCap electronic data capture tools platform^13 14^ and hosted by the University of Oxford.

### Baseline assessments

Following participant consent and urine sample provision, the baseline questionnaire will capture the following information:

1. Whether the participant has previously taken part in the study.
2. Duration of symptoms of the current episode.
3. Participant age, affirmation of female gender assignment at birth and ethnicity.
4. Date and time that urine sample was taken.
5. Symptom severity of the following symptoms on a scale from 0 to 6, where 0 is ‘not affected’ and 6 is ‘as bad as it could be’: Fever, pain or burning when passing urine, increased urgency of urination, increased day and night time frequency of urination.
6. Pregnancy status.
7. Whether the patient suffers from recurrent UTIs.
8. Whether the participant has taken antibiotics in the last 7 days and if so what antibiotics were taken.

Additional information on participants will be collected through a review of each participant’s medical notes by site staff to enable alignment of primary data analysis with the specific inclusion and exclusion criteria defined in POCT manufacturer instructions for use (IFU) documentation, including:

1. Any anatomical or functional abnormalities of the urinary tract.
2. Presence of any indwelling urinary catheters.
3. Whether the participant intermittently self-catheterises.
4. Diagnosis of renal disease.
5. If the participant is classified as immunocompromised according to the following definition: inherited immune disorders; undergoing treatment for cancer; history of haematological malignancy; HIV at all stages; receiving biologics, azathioprine, 6-MP, methotrexate, or ciclosporin; receiving steroids >20 mg/day or less in combination with other immunosuppressive therapies.
6. Diagnosis of diabetes.

The member of staff processing the sample will confirm eligibility of the participant (that the participant is aged 18 or over, female, they have not taken part in the study before and have not had UTI symptoms for longer than 6 days) before they process the sample.

The following information will be collected by site staff during the visit and entered into the eCRF:

1. Time that each POCT and dipstick test was performed.
2. Confirmation from a healthcare professional that a urine sample for analysis is useful for patient care.
3. Whether an antibiotic has been prescribed for this suspected UTI episode, and if so, the class, dose and duration of the antibiotic (this may not be immediately known but will be recorded once known).
4. Results from the POCTs and dipstick tests.
5. Confirmation and date that a sample was sent to the study reference laboratory.

The following information will be collected by staff at the NHS-based reference laboratory and entered into the reference laboratory eCRF:

1. Reference test results.
2. Sample tracking details, including date and time the sample was received at the laboratory.

As patients would normally provide a urine sample as part of standard care, the study does not use any additional interventions that could be considered to carry a risk to participants and so adverse event data will not be collected.

### Sample provision and handling, POCT(s) and reference standard

#### Sample provision and handling

Every participant will provide a midstream urine sample to their practice in a standard 30 mL universal container which does not contain sodium borate (Sterilin Polystyrene Universal, ThermoFisher Scientific, UK). Either fresh or previously collected samples will be used provided that they are processed within the time frame specified by the manufacturer of the POCT, but a fresh sample will be preferred if the participant is willing to provide one. POCTs will be carried out by a member of the practice team who has received training on the devices from the manufacturer—that is, mimicking as closely as possible how the test would be used in ‘real life’ primary care. If a sample is required for local laboratory testing as part of clinical management, the urine sample provided by the participant will be split using a no-touch technique. The fraction required by the local laboratory will continue to be processed as a standard clinical sample using routine NHS processes.

The fraction of the sample required for the study reference standard testing will then be sent to the NHS-based reference laboratory in a 30 mL sodium borate container (Sterilin Polystyrene Universal Boric Acid, Thermofisher Scientific, UK). All samples will be destroyed after all laboratory tests have been performed, although bacterial isolates will be retained.

### Index tests

Index tests will be POCTs for UTI, which may incorporate phenotypic AST or molecular determination of uropathogen resistance to antibiotics. Specific details of study POCTs will be included as protocol appendices, with amendments to the protocol and ethical approval concluded prior to introduction of new tests.

POCTs included in the protocol appendix of the application for ethical review were the Sysmex PA-100 AST System (Sysmex Astrego AB, Uppsala, Sweden)^15^ and the Lodestar DX with UTI test panel (Llusern Scientific, Cardiff, UK).^16^ Both technologies were included in the UK National Institute for Health and Care Excellence Health Technology Evaluation HTE7 ‘POCTs for UTIs to improve antimicrobial prescribing: early value assessment’, which recommended further clinical evidence generation prior to reconsideration of these tests for use. The Sysmex PA-100 AST POCT is an instrument-based assay with a disposable panel for the diagnosis of UTI and phenotypic AST directly from patient urine samples. The PA-100 AST determines bacteriuria within approximately 15 min against a cut-off value of 5×10^4^ colony-forming units (CFU)/mL; if bacteriuria is detected, the analyser continues to determine antimicrobial susceptibility of uropathogens against a panel of antibiotics including amoxicillin-clavulanic acid, ciprofloxacin, fosfomycin, nitrofurantoin and trimethoprim, with the AST step taking approximately 15–30 min. The Llusern Scientific POCT is an instrument-based assay for the loop-mediated isothermal amplification detection of clinically relevant levels of a panel of uropathogens directly from patient urine samples through detection of uropathogen DNA. Target uropathogens include *Escherichia coli*, Enterococcus spp., *Staphylococcus saprophyticus*, *Pseuodomonas aeruginosa*, *Proteus mirabilis* and Klebsiella spp., with the assay taking 40 min to complete following assay initiation. Additional tests may be added beyond those described in this paragraph.

POCT manufacturers will provide training on device use either in person or via video link directly to study sites and to members of the TOUCAN central trial team. Study sites will be asked to adhere to manufacturer prescribed Quality Control schedules and procedures, with instruction provided during device training and through associated instrument manuals.

### Conventional urinalysis (dipstick)

Sites will be provided with 10-parameter urinalysis reagent test strips (RS10, SureScreen Diagnostics, Annesley, UK), incorporating nitrite and leukocyturia detection. This test will be conducted concurrently with POCT runs and interpreted blind to any POCT results. The diagnostic performance of the urine dipstick in relation to the NHS-based reference laboratory result will be assessed and also compared with the diagnostic performance of POCTs. Clinicians caring for the patient will have access to the dipstick test result, which may help direct care.

### Laboratory reference standard

Reference testing will be conducted at the laboratory of the Specialist Antimicrobial Chemotherapy Unit, Public Health Wales. All urine samples will undergo automated microscopy using a Sysmex UF 5000 system, then 50 µL spiral plated onto UTI chromogenic media (diluted according to opacity of urine) and incubated overnight. Bacterial growth will be assessed as pure or predominant uropathogen growth and quantified to CFUs per mL (CFU/mL). Each significant uropathogen will be identified using the MALDI-ToF mass spectrometry method. The UTI definitions set out in the UK Health Security Agency (Public Health England at time of publication) guide on investigation of urine (B41)^11^ will be used for microbiological UTI diagnosis as primary reference standard. The guide defines UTI as either probable or possible UTI according to the following criteria: Probable UTI is defined as ≥10^5^ CFU per millilitre (CFU/mL) of pure culture, irrespective of white blood cell (WBC) levels. Possible UTI is defined using the following criteria: reculture at ≥10^5^ with predominant growth (where the second or third organisms are at least 3×log_2_ CFU/mL below the predominant organism) irrespective of WBC, growth of 2 organisms (dual culture: where both are ≥10^5^ or one is ≥10^5^ and the other ≥10^4^) accompanied by WBC, pure or predominant culture at 10^4^–10^5^ accompanied WBC, predominant culture at 10^4^–10^5^ of a UTI pathogen species accompanied by WBC OR pure or dual culture (where both present are known urinary pathogens at 10^3^–10^4^ accompanied by WBC).

Quantitative culture will allow the application of European Guideline thresholds (≥10^3^ CFU/mL) or similar if appropriate or desired. Antimicrobial susceptibilities will be performed by broth microdilution, the ‘gold-standard’ method, according to the International Standard 20776-1.^12^ Susceptibility to a panel of antibiotics, according to pathogen, will be tested, for example, ampicillin, cefoxitin, cefpodoxime, ciprofloxacin, clindamycin, co-amoxiclav, fosfomycin, nitrofurantoin, penicillin, pivmecillinam, teicoplanin, trimethoprim and vancomycin. Bacterial isolates will be stored at −80°C for further analysis.

### Blinding

The majority of candidate POCTs considered for this study will generate results automatically without user interpretation. Should user interpretation be required, the sites will be asked to perform the POCTs requiring user interpretation prior to interpretation of the urine dipstick and before any other POCT automatically reports results; ordering of testing and interpretation will be communicated to sites during the training of the POCT. All samples will be tested on novel POCTs by staff who are not aware of the reference standard result and are also asked to disregard the outcome of the novel diagnostics in the clinical management of the patient since the performance of these tests is still unclear. Reference laboratory staff will be blinded to the results of POCTs carried out within primary care clinics.

### Statistics and data analysis

The statistical aspects of the study relating to the analysis of the primary outcome are summarised here, with details fully described in a statistical analysis plan that will be finalised before any analysis takes place.

### Statistical methods

To determine the diagnostic accuracy of multiple POCTs to detect UTI and determine antibiotic susceptibility if applicable (some tests may only provide a UTI diagnosis) for manufacturer defined antibiotic panels, participants will be cross-classified into 2×2 contingency tables according to:

- For the case of POCT which incorporate both UTI diagnosis and AST
  - Samples determined to be UTI positive according to the POCT and organisms detected as resistant to panel antibiotic (yes/no).
  - Samples determined to be UTI positive according to the laboratory reference standard and organisms detected as resistant to panel antibiotic (yes/no).
- For the case of POCT which incorporate UTI diagnosis only
  - Samples determined to be UTI positive according to the POCT (yes/no).
  - Samples determined to be UTI positive according to the laboratory reference standard (yes/no).

This information will be used to estimate the sensitivity, specificity, positive and negative predictive values, and positive and negative likelihood ratios for detection of UTI and for the AST where applicable (either phenotypic or molecular), for each POCT. Results will be presented with exact 95% CIs. The differences in the sensitivity and the specificity of the POCT to detect UTI and the corresponding parameters for the urine dipstick will be calculated and presented with 95% CIs. This information will be used to estimate the proportion of samples that would be correctly classified by the POCT but not by the dipstick or vice versa. Results of AST will be presented stratified by organism (as detected by the laboratory reference standard) and antibiotic, with allowance made for organism/antibiotic combinations in which the uropathogen is known to have intrinsic resistance or for which AST is not recommended to be reported in international guidance. AST results will additionally be classified and presented in terms of categorical agreement.

If feasible, for participants whose samples have been tested with more than one comparable POCT (ie, paired sampling), results from the different POCTs will be additionally cross-tabulated against each other. The number of participants prescribed discordant antibiotics will be tabulated and expressed as a proportion of the total, with a 95% CI. These will also be tabulated by the reason for the discordance—for example, an antibiotic prescription without confirmed UTI, or a specific antibiotic being prescribed to an individual for whom the organism was not susceptible to that antibiotic. An exploratory analysis will summarise the baseline symptoms and dipstick results of (1) participants for whom the POCT and laboratory reference test gave discrepant results and (2) participants for whom discordant antibiotic prescriptions were issued. If there are sufficient samples with multiple POCT results available, we will investigate using latent variable methods to simultaneously take account of all results performed on the same sample and quantify any changes of estimates in diagnostic accuracy parameters.^17^

The primary analysis for each POCT assessed will be based on participants’ compatibility with the exclusions of the relevant manufacturer’s IFU and associated Approved Documentation, and a sensitivity analysis will include all participants. The primary analysis will use the laboratory reference standard definition of probable UTI as defined in the details of the reference standard and a sensitivity analysis will use the definition of possible UTI. Another sensitivity analysis will exclude samples detected by laboratory culture to be positive for bacterial species as either the sole or the predominant species where the POCT does not test for this organism. Further sensitivity analyses will be prespecified in the statistical analysis plan.

As most missing test results are likely to be incidental, the primary analysis will use complete-sample data. We will report the test failure rate, with reasons if known, and the proportion of participants who could not be recruited because the time between sample production and analysis is longer than recommended by the manufacturer. To assess whether missing test data biases the diagnostic accuracy assessment,^18^ the distributions of baseline characteristics and POCT results will be compared for individuals with missing results and individuals with non-missing results to check for possible differential patterns of verification. If the proportion of missing data exceeds 10%, we will supplement the primary sensitivity and specificity results with values of the ‘test ignorance region’, that is, the range of sensitivities and specificities that are consistent with the complete data, allowing for non-ignorable missing data.^19^

Where appropriate, results will be presented according to the Standards for Reporting of Diagnostic Accuracy Studies (STARD) guidelines for reporting diagnostic studies.^20^

### Sample size

Sample size requirements for POCTs are based on the estimation of the sensitivity of a POCT to detect reference laboratory culture-confirmed UTI, and key antibiotic resistance markers where applicable. As candidate POCTs may have different levels of diagnostic performance, we present different scenarios, corresponding to sensitivities of 85%, 90%, 95% and 99%. The figures presented here are indicative only and an appropriate choice of sample size may differ, depending on the target performance of the POCT under consideration.

Table 1 relates to testing the sensitivity of the POCT against a fixed target sensitivity, and table 2 indicates the expected precision of the estimated sensitivity for the same range of sample sizes. Test specificity is expected to be estimated with greater precision than test sensitivity, as a majority of samples collected are expected to test negative for UTI.

**Table 1:** Number of ‘positive’ samples required to detect a difference between the assumed sensitivity of the test (rows) and the minimum target sensitivity (columns) with 90% power (first number in each cell) and 80% power (second number in each cell), two-sided test at the 5% significance level

		Minimum target sensitivity		
		0.8	0.85	0.9
Assumed sensitivity of test	0.85	617/471	–	–
	0.9	137/108	471/363	–
	0.95	51/42	96/79	301/239
	0.99	24/21	35/32	64/56

**Table 2:** Expected total width of the 95% CI for the sensitivity, given the sensitivity of test (rows) and the number of ‘positive’ samples (columns)

		Number of positive samples				
		50	100	200	400	600
Assumed sensitivity of test	0.85	0.22	0.15	0.10	0.07	0.06
	0.9	0.19	0.13	0.09	0.06	0.06
	0.95	0.14	0.10	0.07	0.05	0.04
	0.99	0.09	0.06	0.04	0.02	0.02

The numbers in table 1 refer to the numbers of ‘positive’ samples required. In the case of POCTs that attempt to detect UTI, the total sample size is estimated by dividing the appropriate number in the table by the assumed prevalence of UTI. For example, in the case in which the assumed sensitivity is 99%, the minimum target sensitivity is 90%, and the prevalence of UTI is 30% (0.3), the total number of samples required for 90% power is 64/0.3=213. For tests that perform AST, the total sample size is estimated by dividing by the product of the assumed prevalence of UTI and the assumed proportion of UTI samples that contain a pathogen resistant to the antibiotic(s) of interest. For example, in the case in which the assumed sensitivity is 99%, the minimum target sensitivity is 90%, the prevalence of UTI is 30% (0.3) and the proportion of UTI samples that are trimethoprim-resistant is also 30% (0.3), the total number of samples required for 90% power is 64/(0.3×0.3)=711. These assumptions are informed by previously published data for prevalence of laboratory-confirmed UTI and antibiotic resistance in symptomatic patients in primary care settings.^5^

Any assessment of early-stage POCTs will use an initial pilot that aims to recruit 30 positive samples. For UTI detection without AST determination, this would require 100 samples in total, assuming a prevalence of 30%. In this scenario, the pilot study would be expected to estimate sensitivity with a 95% CI total width of 0.25 (eg, 0.72 to 0.97), and specificity with a 95% CI total width of 0.16 (eg, 0.80 to 0.96), which will inform a decision on the suitability of the POCT to continue to a fully powered diagnostic accuracy assessment.

Up to 900 participants will be recruited based on the information outlined above. The sample size may require an amendment if additional POCTs are to be added.

### Embedded qualitative study

#### Aims and objectives

To explore the experiences and perceptions of primary care clinic staff involved in the use of new rapid tests for diagnosing UTI, and how these might affect the feasibility of introducing the tests into primary care settings. To gather information about how the primary care clinic staff view the available tests, how they might implement them as part of consultation for patients with suspected UTI and how this impacts their practice.

### Study design

A qualitative methodology is highly appropriate for capturing and exploring people’s experiences and perceptions of phenomena—in this case, new rapid tests for consultations with women attending primary care for suspected UTI—from the perspectives of the practice staff who will be administering the tests. We will conduct semistructured interviews with the practice staff/trained operators. This method of data collection is well suited to capturing experiences and perceptions and has considerable power to explain actions, decisions and processes. A topic guide will be developed from the existing literature and previous experiences of the research team in conducting such research into point-of-care testing.

A general email will be sent from the TOUCAN Research Team to all the primary care clinics who are taking part in the evaluation of the new tests outlining the embedded qualitative study. Clinicians who have been involved in using the new tests will be invited to take part in the interviews. If they are interested in taking part, they will be asked to contact the TOUCAN research team using contact details given.

When clinicians contact the TOUCAN research team, the purpose of the interview study will be explained and further details will be provided as requested. The information leaflet and the consent form will be sent via email or post as necessary. The clinician will also be informed of the options for timing, location and format of the interview. It is anticipated that all the interviews will be conducted via telephone or through an online platform such as Teams. The clinician will have the opportunity to ask any questions, to receive further information and also to decline any further contact. Clinicians will be informed that they will be able to speak freely at the interview without any negative implications or repercussions on employment. Any clinician who chooses not to participate further in the study will be reassured that this will in no way impact on their current or future working.

If the clinician is happy to proceed, an interview will be arranged. For interviews conducted via the telephone or online, the TOUCAN research team will ensure that participants have read and understood the participant information leaflet, have had an opportunity to have all questions and concerns addressed, and that they are willing to give their consent for the interview to proceed. Verbal informed consent using standardised wording from the informed consent form (ICF) will be captured. In these instances, the researcher will complete the ICF with details of the consent and will securely post/email a copy to the participant for their records.

### Sampling

We aim to interview a range of practice staff involved in the use of the new tests across a range of the primary care clinics, for each of the tests under evaluation. We would anticipate conducting at least 4–5 interviews for each of the tests, incorporating views from a varied sample of clinicians, with different involvement in the process, around the potential use and communication of results. Recruitment for the qualitative study will continue until we are able to build up a sufficiently detailed picture of, and explanatory power for the findings around each of the tests. The decision to stop interviewing will be discussed and agreed among members of the research team. Approximately 20–24 interviews will be carried out.

### Analysis

Our analysis of the qualitative data will be pragmatic and enable us to put together a picture of each of the tests as they are being evaluated, which can contribute to the development of the explanatory trial. The interviews with clinicians will be audio-recorded and transcribed. We will use an adapted framework analytical approach.^21 22^ The transcripts will be coded in NVivo and summaries of coding which contain data around the usability and acceptability of the tests, as well as how they fit into the clinical setting, will be transferred into matrices, which will enable the analysis to be shared among the members of the research team. Other thematic material will also be coded and categorised. The ongoing analysis process will be discussed with the research team, and further developed and refined as interviewing and analysis proceed.

## Patient and public involvement

The study team includes a named public coinvestigator who is fully involved in study management and in discussions around prioritising new diagnostics for the platform. A patient and public involvement (PPI) panel consisting of women with lived experience of UTI has advised on the study since inception. They have helped us to draft the patient-facing details to ensure a clear overview of the study can be gained in the reasonably short time available between presentation to the recruiting site and recruitment. They will be involved in dissemination of our findings.

## Ethics and dissemination

Ethical approval for the project has been received from the London Central Research Ethics Committee (23/LO/0371). The original approved protocol incorporated three candidate diagnostic tests including the Sysmex PA-100 AST System, manufactured by Sysmex Astrego AB and the Llusern Scientific Lodestar DX Analyser with UTI test panel. The third diagnostic was not taken forward primarily due to readiness concerns expressed by the manufacturer, so is not mentioned here.

Results will be published in high-impact peer-reviewed journals, with additional project dissemination through presentation at scientific conferences. Project summaries which can be made publicly available will be developed in collaboration with public contributors and provided through, for example, the study website (https://www.phctrials.ox.ac.uk/studies/toucan-platform-for-uti-diagnostic-evaluation). A detailed dissemination plan will be developed by a communications specialist in consultation with our PPI panel before study conclusion. The summary protocol of the study is available through the website of the ISRCTN Registry at https://doi.org/10.1186/ISRCTN80937472 with reference number 80937472.

Applications to access and use study data following completion and publication will be considered by the independent Primary Care Hosted Research Datasets Independent Scientific Committee (PrimDISC) which is hosted by the Nuffield Department of Primary Care Health Sciences at the University of Oxford.
