# Supplementary material for: plaTform fOr Urinary tract infection diagnostiC evAluatioN (TOUCAN): a protocol for a prospective diagnostic accuracy study of point-of-care testing in patients suspected of acute uncomplicated urinary tract infection in primary care clinics in England
Source: BMJ Open. 2025 Jan 30;15(1):e090012. doi: 10.1136/bmjopen-2024-090012 (PMC11792275; doi:10.1136/bmjopen-2024-090012)
Supplement: Supplementary file 1 [file bmjopen-2024-090012.reviewer_comments.pdf]

PEER REVIEW HISTORY

BMJ Open publishes all reviews undertaken for accepted manuscripts. Reviewers are asked to complete a checklist review form and are provided with free text boxes to elaborate on their assessment. These free text comments are reproduced below.

ARTICLE DETAILS

Title (Provisional)

plaTform fOr Urinary tract infection diagnostiC evAluatioN (TOUCAN): a protocol for a prospective diagnostic accuracy study of point-of-care testing in patients suspected of acute uncomplicated urinary tract infection in primary care clinics in England

Authors

Turner, Philip J; Fanshawe, Thomas; Freeman, Jane; Glogowska, Margaret; Hay, Alastair D; Kenealy, Nicola; Llion, Owain; Lowe, Rebecca; Lown, Mark; Moore, Michael; Tate, Valerie; Wilcox, Mark H.; Wootton, Mandy; Butler, Christopher C.; Hayward, Gail

---

VERSION 1 - REVIEW

**Reviewer**	**1**
**Name**	**Holm, Anne**
**Affiliation**	**University of Copenhagen, The Research Unit for General Practice and Section of General Practice, Department of Public Health**
**Date**	**11-Sep-2024**
**COI**	**None**

This is a nicely designed and described diagnostic study. I only have minor comments/suggestions for revisions

The patient population is described in sufficient detail. Since spectrum bias (or variation) is a real problem in the evaluation of diagnostic studies, you may want to collect a bit more information on patient sociodemografics and co-morbidities than described here.

The index tests are not all described since they are included along the way.

The reference standard is described in sufficient detail

The section on blinding is described in sufficient detail. However, I have trouble understanding whether the "order" of tests is because blinding between individual test results is not possible? That should perhaps be stated, since that is usually the case in general practice.

The statistics should probably mention likelyhood ratios rather than positive and negative predictive values.

May I suggest this method once you have results of several simultaneous tests to determine the minimal necessary diagnostics for each individual patient?

https://academic.oup.com/clinchem/article/58/10/1408/5620819

**Reviewer**	**2**
**Name**	**Luchristt, Douglas**
**Affiliation**	**Duke University School of Medicine**
**Date**	**25-Sep-2024**
**COI**	**None**

This is a well written paper and well conceived study, particularly given some of the unknowns of the products that will be tested.

I provide the following recommendations to help strengthen the work.

While all of the information around the definition of UTI and the comparators are located in the paper, it requires digging and would be helpful to more clearly define the primary outcome and be more clear throughout when referencing.

The blinding for the POCT and its potential implications for clinical care is not fully explained and potentially not fully accounted for as this could influence handling of the specimens and other clinical decision making which is an element of the inclusion criteria.

Methodology for analysis of the qualitative portions of this work could be explained more fully and with provided references.

There are many references to GP surgeries. I am not familiar enough with the NHS but am very confused by these statements / references

---

VERSION 1 - AUTHOR RESPONSE

Reviewer: 1
Dr. Anne Holm, University of Copenhagen
Comments to the Author:
This is a nicely designed and described diagnostic study. I only have minor comments/suggestions for revisions

The patient population is described in sufficient detail. Since spectrum bias (or variation) is a real

problem in the evaluation of diagnostic studies, you may want to collect a bit more information on patient sociodemografics and co-morbidities than described here.

*We are very grateful to Dr Holm for her review and for this thoughtful recommendation. Since submission of the original manuscript for review, we amended the study protocol to capture more information with respect to the health status of participants through medical notes review, largely so that we can align primary data analysis with exclusions set out in POCT manufacturer instructions for use documentation. This amendment was approved by the ethics committee and we have updated the manuscript on page 11 to reflect this.

The index tests are not all described since they are included along the way.
The reference standard is described in sufficient detail

*We have added details of the index tests to the manuscript on page 13.

The section on blinding is described in sufficient detail. However, I have trouble understanding whether the "order" of tests is because blinding between individual test results is not possible? That should perhaps be stated, since that is usually the case in general practice.

*We are grateful for this observation and have amended the text in the ‘Blinding’ section on page 15 to disambiguate this process for the reader. Where applicable, we have asked sites to interpret user-interpreted index tests before reading urinalysis dipsticks and prior to the availability of automated results from other POCTs so that users are not influenced in their interpretation of ‘eye read’ assays.

The statistics should probably mention likelyhood ratios rather than positive and negative predictive values.

*We would expect to calculate and report likelihood ratios routinely together with the other described metrics in any results papers, so we have now stated this explicitly in this manuscript on pages 8 and 15.

May I suggest this method once you have results of several simultaneous tests to determine the minimal necessary diagnostics for each individual patient?
https://academic.oup.com/clinchem/article/58/10/1408/5620819

*We are very grateful for this suggestion and will give this our consideration.

Reviewer: 2
Dr. Douglas Luchristt, Duke University School of Medicine
Comments to the Author:

This is a well written paper and well conceived study, particularly given some of the unknowns of the products that will be tested.

*We thank Dr Luchristt for these positive observations and for his review of the manuscript.

I provide the following recommendations to help strengthen the work.

While all of the information around the definition of UTI and the comparators are located in the paper, it requires digging and would be helpful to more clearly define the primary outcome and be more clear throughout when referencing.

*We are grateful for this suggestion to improve the clarity of the manuscript and have added additional context to the ‘Primary objective’ section on page 8 and have referenced the relevant UK guideline and international standard related to the microbiological determination of UTI, definitions of UTI and antimicrobial susceptibility testing.

The blinding for the POCT and its potential implications for clinical care is not fully explained and potentially not fully accounted for as this could influence handling of the specimens and other clinical decision making which is an element of the inclusion criteria.

*The section on blinding on page 15 has been updated for clarity as described in our response to the similar observation of Dr Holm above. We have also added the following sentence to the blinding section to clarify how recruiting site staff are instructed to deal with the results obtained from index POCTs ‘All samples will be tested on novel POCTs by staff who are not aware of the reference standard result and are also asked to disregard the outcome of the novel diagnostics in the clinical management of the patient, since the performance of these tests is still unclear’.

Methodology for analysis of the qualitative portions of this work could be explained more fully and with provided references.

*We are very grateful for Dr Luchristt’s interest in the qualitative elements of the protocol and have updated the ‘Analysis’ section on page 20 with additional detail and with two additional references.

There are many references to GP surgeries. I am not familiar enough with the NHS but am very confused by these statements / references

*We appreciate that terms such as ‘GP surgeries’ are quite UK-specific vernacular so we have amended this descriptor throughout the manuscript to ‘primary care clinics’ and hope this is more broadly understood by a global audience.

Reviewer: 1
If you have selected ‘Yes’ above, please provide details of any competing interests.: None

Reviewer: 2
If you have selected ‘Yes’ above, please provide details of any competing interests.: None

VERSION 2 - REVIEW

**Reviewer**	**1**
**Name**	**Holm, Anne**
**Affiliation**	**University of Copenhagen, The Research Unit for General Practice and Section of General Practice, Department of Public Health**
**Date**	**13-Dec-2024**
**COI**	

Thank you for your revisions of the manuscript, which is very much improved and, to my opinion, ready for publication

---

**Reviewer**	**2**
**Name**	**Luchristt, Douglas**
**Affiliation**	**Duke University School of Medicine**
**Date**	**02-Jan-2025**
**COI**	

Thank you for the revisions in response to my and other reviewer's comments.
